# Increased endogenous PKG I activity attenuates EGF-induced proliferation and migration of epithelial ovarian cancer via the MAPK/ERK pathway

**DOI:** 10.1038/s41419-023-05580-y

**Published:** 2023-01-19

**Authors:** Ting Lan, Ying Li, Yue Wang, Zhong-Cheng Wang, Chun-Yan Mu, Ai-Bin Tao, Jian-Li Gong, Yuan Zhou, Hao Xu, Shi-Bao Li, Bing Gu, Ping Ma, Lan Luo

**Affiliations:** 1 Xuzhou Key Laboratory of Laboratory Diagnostics, Xuzhou Medical University, Xuzhou City, Jiangsu Province China; 2 School of Medical Technology, Xuzhou Medical University, Xuzhou City, Jiangsu Province China; 3 Department of Laboratory Medicine, Affiliated Hospital of Xuzhou Medical University, Xuzhou City, Jiangsu Province China; 4 Division of Cardiology, Department of Medicine, The Affiliated People’s Hospital of Jiangsu University, Zhenjiang, China; 5 Perlmutter Cancer Center and Department of Surgery, NYU Langone Health, New York, NY USA; 6 Department of Gynecology Huangshi Love & Health Hospital affiliated to Hubei Polytechnic University, Hubei City, Wuhan Province China

**Keywords:** Oral cancer, Proteins

## Abstract

The type I cGMP-dependent protein kinase (PKG I) is recognized as a tumor suppressor, but its role in EGFR regulated epithelial ovarian cancer (EOC) progression remains unclear. We evaluated the in vivo and in vitro effects of activated PKG I in EGF-induced EOC cell proliferation, migration, and invasion. The expressions of EGFR and PKG I were elevated, but the activated PKG I was decreased in EOC tissues of patients and cells lines. The addition of 8-Br-cGMP, a specific PKG I activator, attenuated the EGF-induced EOC cell proliferation, migration, and invasion in vitro. Similarly, activated PKG I also attenuated EOC progression in vivo using an EOC xenograft nude mouse model. The activated PKG I interacted with EGFR, causing increased threonine (693) phosphorylation and decreased tyrosine (1068) phosphorylation of EGFR, which resulted in disrupted EGFR-SOS1-Grb2 combination. Subsequently, the cytoplasmic phosphorylation of downstream proteins (c-Raf, MEK1/2, and ERK1/2) were declined, impeding the phosphorylated ERK1/2’s nucleus translocation, and this reduction of phosphorylated tyrosine (1068) EGFR and ERK1/2 were also abolished by Rp-8-Br-cGMPS. Our results suggest that the activation of PKG I attenuates EGF-induced EOC progression, and the 8-Br-cGMP-PKG I-EGFR/MEK/ERK axis might be a potential target for EOC therapy.

## Introduction

In 2018, there were an estimated 295,414 new cases and 184,799 deaths from ovarian cancer globally, ranking the 8th most common cancer and cause of death for females [1]. Ovarian cancer is classified by the cells of origin as epithelial (90%), stromal (5–6%), and germ cell tumors (2–3%) [2]. The most common type of epithelial ovarian cancer (EOC) usually presents with no specific symptoms, making it mostly diagnosed at late-stage with a low 5-year survival rate of 29% [3, 4]. In contrast, few early-staged patients (15%) have a markedly high rate of 92%, implying the need to understand EOC’s etiology. EOC comprises heterogeneous histological subtypes with unique molecular features. Mutations in breast cancer susceptibility genes *BRCA1/2* and other genes like *CDH1*, *PALB2*, *PTEN*, and *TP53* have been identified as causative factors in hereditary EOC [5, 6]. The poly (ADP-ribose) polymerase (PARP) mediates DNA repair in *BRCA1/2-*mutated cells facilitating its survival [7]. Thus, PARP inhibitors have been tested clinically achieving therapeutic benefits, especially for *BRCA*-mutated EOC. But inconsistent results are yielded in patients without *BRCA* mutation [8]. Apart from the PARP inhibitors, anti-angiogenesis agents have also generated positive outcomes in randomized phase III trials, associated toxicity and adverse events are reported yet [9]. Therefore, novel therapy target discovery is imperative to improve EOC outcome and mortality.

Epidermal growth factor receptor (EGFR) is a transmembrane glycoprotein and a member of the cell membrane receptor tyrosine kinase family [10]. EGFR binds with its cognate ligands (EGF, etc.), promoting carcinogenesis via cell proliferation, migration, and invasion [11]. EGFR is overexpressed in all histological EOC subtypes, ranging from 9 to 62% varied with the antibody and cutoff value [12–14]. Overexpressed EGFR is associated with the late-stage and high patient mortality in EOC [14, 15]. Nevertheless, discouraging results have been reported in clinical trials using EGFR-targeted agents [16]. And EGFR seems to have a less role in prognostic evaluation of EOC [12, 17]. For this reason, it warrants profound insights into identifying the mechanisms of resistance to anti-EGFR therapies and the role of EGFR in EOC.

cGMP-dependent protein kinase G (PKG) is a serine/threonine kinase widely expressed in mammalian cells and contains two major types that soluble PKG I and membrane-associated PKG II. Upon binding to cGMP, PKG I is activated and phosphorylates targeted proteins at serine/threonine residues regulating downstream effects. The activation of PKG I is involved intestinal secretion, bone growth, neuronal signaling, and cell apoptosis [14, 18, 19]. Recently, emerging studies have suggested an anti-tumor effect of activated cGMP/PKG signaling in multiple cancer types via inhibiting cell growth, metastasis, and immunity [20]. Thus, the cGMP/PKG pathway may serve as a potential target for cancer treatment. Traci et al. found that sGC stimulators and PDE5 inhibitors increased cGMP levels, reducing cell viability and apoptosis in head and neck squamous cell carcinoma [21]. Fallahian et al. reported that the PKG I was sufficient to induce apoptosis in estrogen receptor-positive and negative breast cancer cell line [22]. The function of PKG I in ovarian cancer is controversial. Some research reported PKG Iα activity significantly induced DNA synthesis and cell proliferation [23]. While others indicated that PKG I inhibited cell proliferation in ovarian cancer [24]. Notably, the role of PKG I in EGFR-related EOC progression remains unclear.

In the present study, we first examined the expressions of PKG I and EGFR in EOC tissues and cells. Then we tested whether the 8-Br-cGMP, PKG I activator, could affect the EGF-induced EOC cell growth and metastasis in vivo and in vitro. Last, we investigated the underlying mechanisms of activated PKG I in attenuating EGF-induced EOC progression.

## Materials and methods

### Reagents and antibodies

Antibodies against PKG I, EGFR, p-EGFR (T693), ERK1/2, VASP, c-Raf, MEK1/2, p-VASP (Ser 239), p-c-Raf (Ser338), p-Erk1/2 (Thr202/Tyr204), MEK1/2 (Ser217/Ser221), and Grb2 were purchased from Affinity Biosciences (Affinity Biosciences, OH, USA). Antibodies against p-EGFR (Tyr1068) was purchased from Cell Signaling Technology (Danvers, MA, USA). Antibodies against Sos1 was purchased from Santa Cruz (Santa Cruz, CA, USA). Antibodies against p-Thr/ser was purchased from Abcam Biotechnology (Abcam, Cambridge, UK). Antibody against β-actin was purchased from Proteintech (Proteintech, Chicago, USA). EGF was purchased from PeproTech (Rocky Hill, NJ, USA), 8-Br-cGMP, and Rp-8-Br-cGMPS were purchased from Biolog Biotechnology (Bremen, Germany) (Table 1).

### Tissue specimens and cell culture

A total of 30 pairs of EOC tissues and the adjacent normal controls were collected from the Affiliated Hospital of Xuzhou Medical University from 2019 to 2021. The tissues were frozen at −80 °C until further use. All the patients were all diagnosed as EOC for the first time without radiotherapy, chemotherapy, or biological therapy prior to surgery. This study was approved by the Ethics Committee of the Affiliated Hospital of Xuzhou Medical University (XYFY2019-KL113-01).

Human EOC cell lines SKOV3 and A2780, and the human normal epithelial cell line IOSE80 were obtained from American Type Culture Collection (ATCC) with authentication by short-tandem-repeat analyses. The cells were cultured in Dulbecco’s modified Eagle’s medium containing penicillin/streptomycin (DMEM) (Kaiji, Jiangsu, China) with 10% fetal bovine serum (Gibco, Grand Island, NY, USA) at 37 °C under 5% CO_2_.

### ELISA

According to the manual of cGMP ELISA kit (Enzo, Taksim), the cell supernatant (100 µl) was added to the bottom of the appropriate wells, blue conjugate (50 µl) and yellow cGMP antibody (50 µl) were added subsequently, and then incubated at room temperature for 2 h. Clean each well with wash buffer for 3 times. Add 200 µl substrate solution to each well and incubate at room temperature for 1 h. Add 50 µl stop solution and then measure absorbance at 405 nm.

### Western blot

After the indicated treatment (8-Br-cGMP added for 1 h first, and then EGF was added for 5 min), cells were collected and lysed in cell lysis buffer (Kaiji, Jiangsu, China) on ice for 30 min. Subsequently, the supernatant was separated by centrifugation at 14,000 × *g*, 4 °C, 20 min. The protein concentrations were determined with a BCA kit (Kaiji, Jiangsu, China), and protein samples were mixed with 5× sodium dodecyl sulfate (SDS) loading buffer (Beyotime, Beijing, China) before denaturation in the boiling water bath for 10 min. Proteins were separated via 8–12% sodium dodecyl sulfate polyacrylamide gel electrophoresis (SDS-PAGE) and then transferred to polyvinylidene difluoride (PVDF) membranes (Millipore, Bedford, MA, USA). Next, the membranes were blocked with 5% skimmed milk for 2 h at room temperature and then incubated with primary antibodies overnight at 4 °C. The following day, the membranes were incubated with horseradish peroxidase-conjugated secondary antibody for 1 h at room temperature. All bands were detected using the electrochemiluminescence kit and scanned by Bio-Rad (Hercules, CA, USA). β-actin served as an internal control. Image Lab software (4.0) was used for the quantitative analysis of the gray values.

### Cell counting Kit-8 (CCK-8)

Approximately 4 × 10^3^ cells were seeded in 96-well plates in DMEM containing 10% FBS, penicillin/streptomycin. After the indicated treatment (8-Br-cGMP added for 1 h firstly, and then EGF was added for 5 min), 100 µl free medium containing 10 µl CCK-8 solution (Vicmed, Jiangsu, China) was added to each well and incubated for 1 h (37 °C, 5% CO_2_). The optical density values were detected at 450 nm using an enzyme labeling instrument.

### Colony formation

Approximately 500 cells were plated on 6-well plates in DMEM containing 10% FBS, penicillin/streptomycin. About 10–14 days later, visible colonies were fixed by 4% paraformaldehyde for 15 min and then stained with 0.1% crystal violet for 15 min. The units of colony formation were counted using Image J software (1.52 v).

### Wound healing assay

Approximately 5 × 10^6^ cells were seeded in 6-well plates in DMEM containing 10% FBS penicillin/streptomycin. Next day, a 10 µl tip was used to scratch the cells in the plates. And the cells were washed three times with PBS to remove cell debris. Then cells were treated with serum-free medium and the indicated compounds. Microphotographs were taken at 0, 24, and 48 h after wounding. The wound area was calculated by manually tracing the cell-free area in the captured images using Image J software (1.52 v).

### Trans-well migration and invasion assay

For the migration assay, 4 × 10^4^ cells were seeded into the upper chambers of the trans-well plates (8 µm pore size; Corning, NY, USA) in 200 µl serum-free culture medium, whereas 600 µl of DMEM medium supplemented with 10% FBS was added to the lower compartments of the chambers. For the invasion assay, the chambers were coated with a thin layer of Matrigel before the cells were seeded. After 24 or 48 h of incubation at 37 °C in a 5% CO_2_ incubator, the cells remaining in the upper chamber were removed carefully with cotton swabs. The cells migrated or invaded to the bottom of the chamber were fixed with 4% paraformaldehyde solution for 15 min and stained with a 0.1% crystal violet solution for 10 min. Finally, the stained cells were counted in five randomly selected fields per membrane under an inverted light microscope.

### Flow cytometry

The Annexin V/APC staining kit (Kaiji, Jiangsu, China) was used to analyze apoptosis. Briefly, 1 × 10^5^ cells were suspended in 100 µl buffer, then, 5 µl Annexin V-FITC and 5 µl Propidium Iodide were added. After incubation for 10 min at room temperature, the cells were analyzed by flow cytometer within 1 h.

### Immunofluorescence

Approximately 1 × 10^3^ cells in the 24-well plates were fixed with 4% paraformaldehyde at room temperature for 15 min. Then, the cells were permeabilized with 0.3% Triton-X 100 for 10 min and incubated with 5% BSA for 1 h at room temperature. The cells were incubated with the indicated primary antibodies at 4 °C overnight and then with the corresponding secondary antibodies avoiding light at room temperature for 1 h next day. Subsequently, 200 µl DAPI (Beyotime, Beijing, China) was applied for 10 min at room temperature to stain cell nuclei.

### Immunohistochemistry

Tissues were fixed with 10% neutral formalin and embedded in paraffin. sections were cut serially from paraffin-embedded tissues, placed in a 60 °C incubator for 1 h. Antigen retrieval was implemented using sodium citrate antigen retrieval solution (pH 6.0) through microwave method after dewaxing and rehydration with xylene and gradient ethanol, and endogenous per-oxidase was removed using 3% H_2_O_2_. After blocking with the 3% BSA for 30 min, the slices were incubated with the first antibody overnight at 4 °C. On the next day, the sections were washed with PBS to incubate with second antibodies for 60 min. Finally, sections were stained by diaminobenzidine tetrahydrochloride (DAB) and hematoxylin staining. Images were observed under multifunction microscope (Olympus, Tokyo, Japan) Scoring was performed blindly by two pathologists.

### Coimmunoprecipitation and Immunoprecipitation

The lytic cells were transferred to microcentrifuge tubes using Western and IP lysate (Kaiji, Jiangsu, China), and centrifuged at 140, 000 × *g* for 20 min at 4 °C. The supernatants were transferred to another EP tube and incubated with primary antibodies overnight at 4 °C with agitation. The second day, the Magnetic beads (MedChemExpress, New Jersey, USA) were added to the EP tube and stirred for 2 h at 4 °C. Then, the EP tubes were placed in magnetic racks, adsorbed for 2 min, washed for four times with pre-cooled PBS, and finally an appropriate amount of protein loading buffer was added to each tube and boiled at 100 °C for 5 min. Samples were used for Western blot.

### Mass spectrometry (MS)

To identify phosphorylation sites, EGFR was immunoprecipitated from the cellular extract of ~5 × 10^7^ SKOV3 and A2780 cells which were serum-starved for 12 h and then treated with 8-Br-cGMP (250 mM) for 1 h. Afterward, the precipitated samples underwent on-bead enzymatic digestion. The phosphor-peptides of the digestion products were enriched by Tidoped mesoporous silica (Ti-MPS) and then subjected to Nano LC-MS/MS according to reference [14]. The acquired MS raw files were analyzed by MaxQuant environment (version 1.2.2.5). The tandem MS was searched against the UniProt proteome (version 20120418). Enzyme specificity was set to trypsin (KR/P). Carbamidomethyl (C) was chosen for fixed modifications and oxidation on methionine, the phospho (S, T, Y) was set as variable modifications. The false detection rates for peptides, proteins, and phosphorylation were all set below 0.01. All the other parameters in MaxQuant were set to default values.

### Xenograft experiments

Immunodeficient 4–6-week-old female mice (BALB/c-nu, weighing 18–21 g, Shanghai Experimental Animal Center of the Chinese Academy of Sciences) were included in this study. This study was approved by the Institutional Animal Care and Use Committee of Xuzhou Medical University (202005A009). All animal procedures were performed following the institutional and national guidelines.

A single A2780 cells (5 × 10^6^ cells suspended in 200 µl PBS) solution was inoculated subcutaneously in the right hypochondrium of mice. When the tumor grew to about 1 cm^3^ in size, the nude mice were sacrificed. The subcutaneous tumor was removed and digested with 0.125% trypsin for 8–10 times. Then the supernatant was collected, filtered with a 200-mesh cell filter, and cultured in a petri dish. After 4 to 5 passages, A2780 cells (5 × 10^6^ cells suspended in 200 µl PBS) were intraperitoneally injected in the nude mice. The next day, the mice were randomly divided into three groups (*n* = 5) and intraperitoneally injected with 8-Br-cGMP (0, 25, or 50 mg/kg per mouse, every two days until day 14). After about 2 weeks, all the nude mice were sacrificed, and the liver and tumor were collected for further analysis. All animals were randomized and blinded for experimental grouping.

### Statistical analysis

All data were presented as the mean ± SD. The statistical significance was performed by Student’s t test between two groups, or by the one-way or two-way analysis of variance (ANOVA) followed by the Turkey’s multiple comparisons test among groups (GraphPad Prism 9.3.0). *p < 0.05; **p < 0.01; ***p < 0.001; ****p < 0.0001 were considered statistically significant.

## Results

### The expression of PKG I was elevated, but its activity was downregulated in EOC tissues and cell lines

**Fig. 1:**
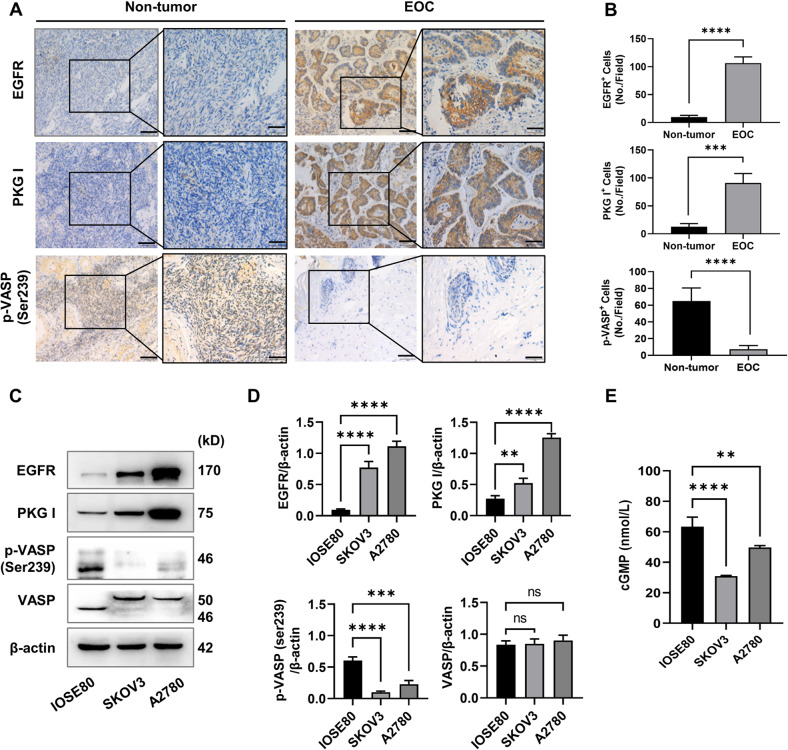
The phosphorylation levels of VASP (p-VASP) and cGMP are significantly decreased in epithelial ovarian cancer. Represent immunohistochemistry (IHC) images (**A**) and quantitative data the number of high expression cells (**B**) on the EGFR, PKG I, and p-VASP (Ser239) in 30 paired adjacent normal and tumor tissues of EOC patient. Error bars represent the means ± SD. Scale bar: 100 μm (left), 50 μm (right). Quantitative data on the expressions (**C**, **D**) Western blot analysis the expressions of EGFR, PKG I, p-VASP (Ser239), and VASP in EOC cells (SKOV3 and A2780) and normal ovarian epithelial cells (IOSE80). **E** ELISA analysis the cGMP concentrations in SKOV3, A2780, and IOSE80 cells. All data are presented as mean ± SD (*n* = 3 to 5 experiments). Significance was evaluated by Student’s t test in (**B**) and Ordinary two-way ANOVA analysis followed by Tukey’s multiple comparisons test in (**D**, **E**). *p < 0.05; **p < 0.01; ***p < 0.001; ****p < 0.0001 under indicated comparison.

We first confirmed that the EGFR was overexpressed in EOC patient ovary tissues by IHC analysis (Fig. 1A, B), consisting with the previous studies [25]. We then investigated the expression of PKG I and its activity by the phosphorylation of Vasodilator Stimulated Phosphoprotein (VASP) at serine 239 [26]. The expression of PKG I was elevated, but the phosphorylation of VASP at serine 239 (p-VASP) was downregulated in EOC patient ovary tissues (Fig. 1A, B). In addition, we also found elevated EGFR and PKG I expression and declined p-VASP expression in EOC cells (SKOV3 and A2780) than that of normal ovarian epithelial cells (IOSE80) (Fig. 1C, D). PKG I is activated by the second messenger cGMP. The declined cGMP levels in SKOV3 and A2780 cells than the IOSE80 cells may account for their declined PKG I activity (Fig. 1E).

### Activation of PKG I attenuated EGF-induced EOC cell proliferation, migration, and invasion in vitro

**Fig. 2:**
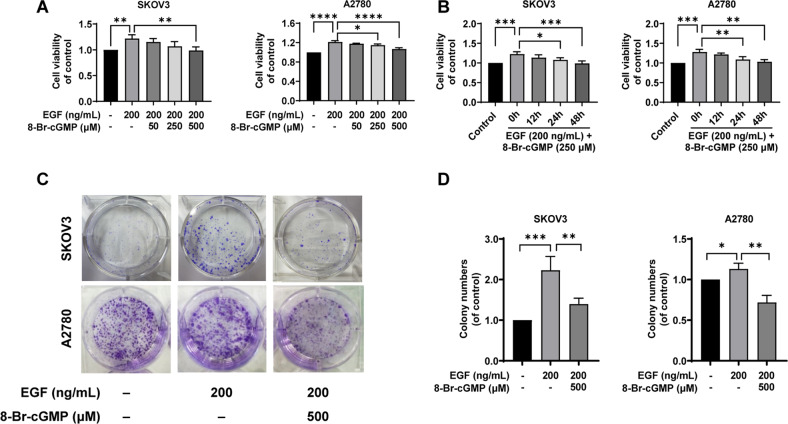
Activation of PKG I attenuated EGF-induced EOC cell proliferation in vitro. **A**, **B** EOC cell proliferation was analyzed by CCK8 assays. **C**, **D** R Representative results of colony formation; the number of colonies counted was of an entire well and the error bars represent mean ± SD from three independent experiments. Significance was evaluated Ordinary two-way ANOVA analysis followed by Tukey’s multiple comparisons test in (**A**, **B**, **D**). *p < 0.05; **p < 0.01; ***p < 0.001; ****p < 0.0001 under indicated comparison.

Uncontrolled proliferation is a major feature of cancer cells. We first found the 24h-EGF treatment (200 ng/mL) significantly increased the cell viability of SKOV3 and A2780 cells using the CCK-8 assay (Fig. 2A). We also found that the 8-Br-cGMP (0, 50, 250, 500 µM) supplement, a specific PKG I activator, upregulated the expression of p-VASP in a dose-dependent manner (Fig. 1A, B). Significantly, EGF-induced EOC cell proliferation was attenuated by the 8-Br-cGMP (0, 50, 250, 500 µM) supplement dose-dependently, and when 8-Br-cGMP increased to 250 µM, cell viability began to be inhibited, about 0.8- and 0.9-fold of the EGF group in SKOV3 and A2780. Therefore, 250 µM was selected for assessing the effect of time on the cell viability (Fig. 2A). We also assessed whether the 8-Br-cGMP supplement could inhibit EOC cell proliferation continuously. The cell viability of EOC cells was evaluated after treatment with EGF (200 ng/mL) and 8-Br-cGMP (250 µM) for 0, 12, 24, and 48 h. The cell viability of SKOV3 and A2780 cells was significantly decreased at 24 h and 48 h compared with that of 0 h culture (Fig. 2B). We then confirmed the effect of activated PKG I on EOC cell proliferation by the clone formation assay. In the CCK-8 experiment, the inhibitory effect was best when 500 µM 8-Br-cGMP was added. Therefore, we chose 500 µM in the colony formation assay (Fig. 2C). The EGF treatment (200 ng/mL)-induced high number and area of clone formation in SKOV3 and A2780 cells were significantly suppressed by the 8-Br-cGMP supplement (500 µM), and after adding 8-Br-cGMP, the number of cells decreased by 40% and 45% in SKOV3 and A2780 cells, respectively (Fig. 2D).

**Fig. 3:**
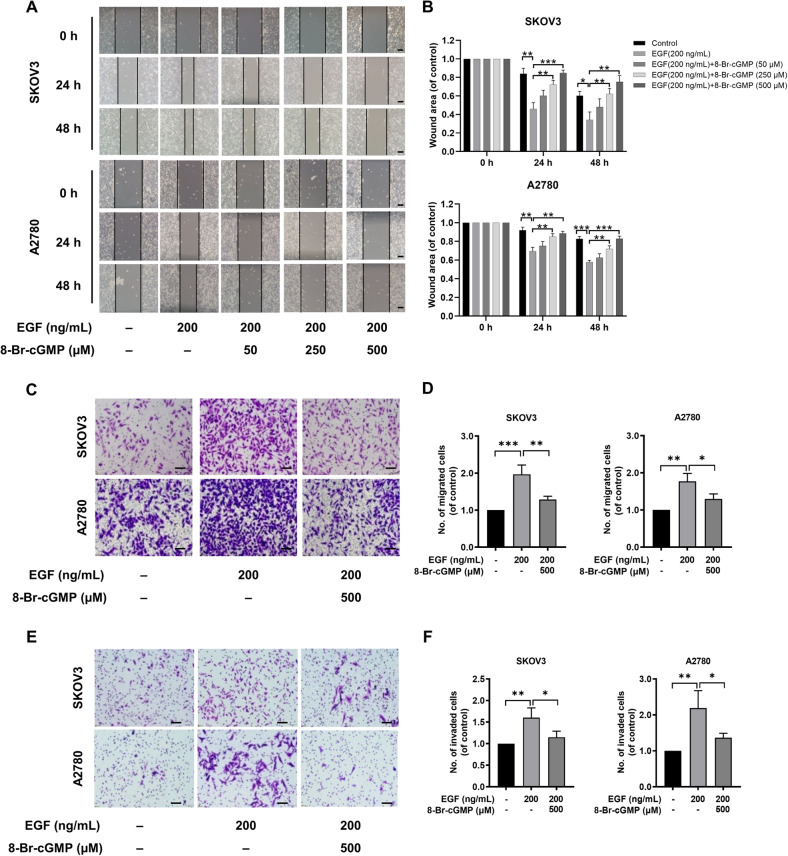
Activation of PKG I attenuated EGF-induced EOC cell migration and invasion in vitro. **A**, **B** Representative results of wound healing assays. The bar chart represents the migration distance. Error bars represent the means ± SD of three independent experiments (5 fields/experiment). **C**, **F** Representative images Transwell assay was used to detect the effect of PKG I activation on the migration and invasion ability Scale bar, 50 μm. Quantitative data on the migrated and invaded SKOV3 and A2780 cells of Trans-well assay. The data were presented as mean ± SD (*n* = 3). The bar chart represents the migration and invasion cell numbers. Error bars represent the means ± SD of three independent experiments (5 fields/experiment). Significance was evaluated Ordinary two-way ANOVA analysis followed by Tukey’s multiple comparisons test in (**B**, **D**, **E**). *p < 0.05; **p < 0.01; ***p < 0.001 under indicated comparison.

Next, we examined the effect of activated PKG I on EOC cell migration and invasion. The EGF treatment (200 ng/mL) significantly increased the cell migration of SKOV3 and A2780 cells using the wound healing (Fig. 3A, B) and trans-well assays (Fig. 3C, D). Similarly, the 8-Br-cGMP supplement markedly suppressed EGF-induced cell migration in SKOV3 and A2780 cells dose-dependently at 24 h and 48 h (Fig. 3A, B). And this was further proved by the trans-well assay (Fig. 3C, D). However, there was no statistical difference in inhibition of 8-Br-cGMP on cell migration at 24 h and 48 h in the wound healing. The EGF-induced cell invasion in SKOV3 and A2780 cells were also blocked by the 8-Br-cGMP (500 µM) supplement (Fig. 3E, F).

Activated PKG I also inhibited ovarian cancer proliferation (Fig. 2A), invasion (Fig. 2B), and metastasis (Fig. 2C) in the absence of EGFR activation, but the effect was less significant than that of EGF-stimulated therapy. Activated PKG I had no effect on the proliferation of normal ovarian epithelial cells (Fig. 2A). Therefore, our experiment was based on EGF activation status of EGFR, and confirmed that activation of PKG I effectively inhibited the migration and invasion of ovarian cancer cells. In addition, it showed that activation of PKG1 had no effect on apoptosis of ovarian cancer cells (Fig. 2D, E).

**Fig. 4:**
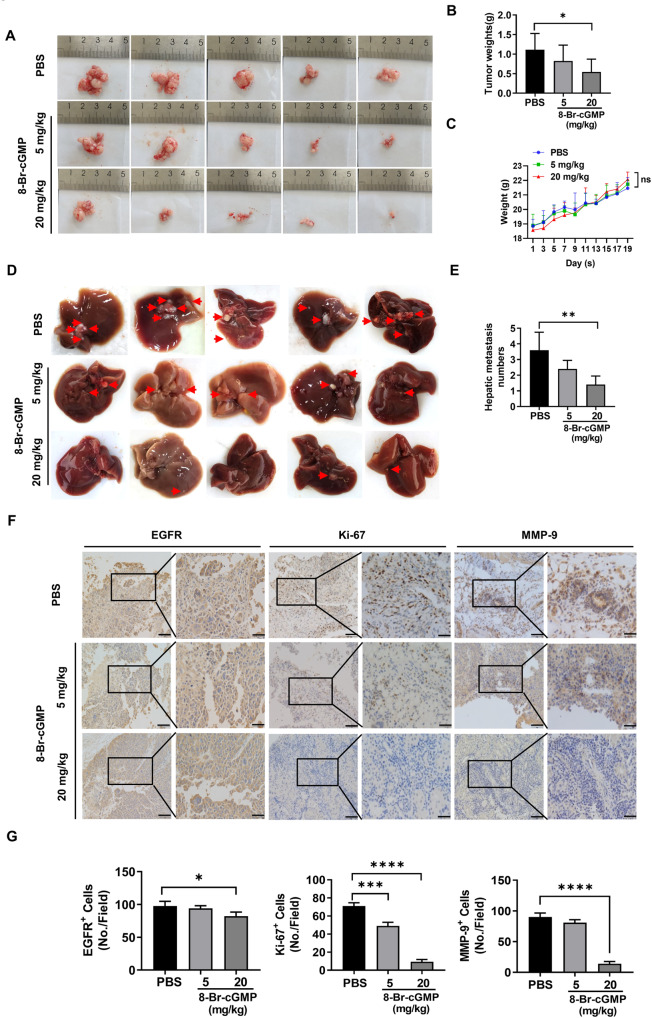
Activation of PKG I suppressed tumorigenesis and metastasis in vivo using an EOC xenograft model. **A** The xenograft models were generated after injected with A2780 cells and the administration of PBS or 8-Br-cGMP (5 or 20 mg/kg) in nude mice (*n* = 5/group). **B** The tumor weight was measured after the nude mice were euthanized. Error bars represent the means ± SD. **C** Weight changes of nude mice in different groups. **D** Images of hepatic metastasis (indicated by the red arrow) in nude mice injected with A2780 cells and the administration of PBS or 8-Br-cGMP (5 or 20 mg/kg). **E** The number of hepatic metastasis was counted after nude mice were euthanized. Error bars represent the means ± SD. **F** IHC analysis of EGFR, Ki-67, and MMP-9 in tumor tissues of nude mice. Scale bar: 100 μm (left), 50 μm (right). **G** Quantitative data on the expressions of EGFR, Ki-67, and MMP-9 in tumor tissues of nude mice. The results were presented as mean ± SD (*n* = 3). Significance was evaluated Ordinary two-way ANOVA analysis followed by Tukey’s multiple comparisons test in (**B**, **E**, **G**) and Two-way ANOVA followed by Tukey’s multiple comparisons test in (**C**). *p < 0.05; **p < 0.01; ***p < 0.001; ****p < 0.0001 under indicated comparison.

Rp-8-Br-cGMPS, the antagonist of 8-Br-cGMP, was a PKG I inhibitor. So, we investigated whether Rp-8-Br-cGMPS could reverse the effect of activated PKG I. The Rp-8-Br-cGMPS treatment (250 µM) indeed reduced the elevated p-VASP expression by 8-Br-cGMP supplement in SKOV3 (Fig. 3A) and A2780 cells (Fig. 3B). Consequently, the inhibition effect of activated PKG I on EOC cell proliferation, migration, and invasion were reversed by Rp-8-Br-cGMPS treatment (Figs. 3, 4). In SKOV3, when treated with the 8-Br-cGMP 24 h, the scratch distance increased compared with the EGF group, and after the addition of Rp-8-Br-cGMPS, the scratch distance again decreased. when treated with 8-Br-cGMP 48 h, the scratch distance increased compared with the EGF group, and after the addition of Rp-8-Br-cGMPS, the scratch distance again decreased. In A2780, after the addition of 8-Br-cGMP 24 h, the scratch distance increased compared with the EGF group, then the addition of Rp-8-Br-cGMPS, the scratch distance again decreased. After the addition of 8-Br-cGMP 48 h the scratch distance increased compared with the EGF group, then the addition of Rp-8-Br-cGMPS, the scratch distance again decreased (Fig. 4). These data suggested that improved PKG I activity could suppress the EGF-induced EOC proliferation, migration, and invasion in vitro.

### Activation of PKG I suppressed tumorigenesis and metastasis in vivo using an EOC xenograft model

Next, we speculated that activation of PKG I could suppress tumor growth and metastases in vivo using an EOC xenograft mouse model. A2780 cells were intraperitoneally injected into the nude mice, and 8-Br-cGMP (5 or 20 mg/kg) or PBS as control was administered the next day. After three weeks, the average volume (Fig. 4A) and weight (Fig. 4B) of the abdominal tumor were lower in mice injected with 8-Br-cGMP (20 mg/kg) than the control. There was no statistically significant difference in body weight between the administration and control groups (Fig. 4C). We also counted the tumor number that metastatic to the liver. The 8-Br-cGMP administration inhibited EOC’s liver metastasis in a dose-dependent manner (Fig. 4D, E). Compared with the control group, 8-Br-cGMP-treated mice showed lower expression of EGFR, Ki-67, and MMP-9, thus inhibiting tumorigenesis and metastasis (Fig. 4F, G).

### Activated PKG I bound with EGFR leading its threonine 693 site phosphorylation

**Fig. 5:**
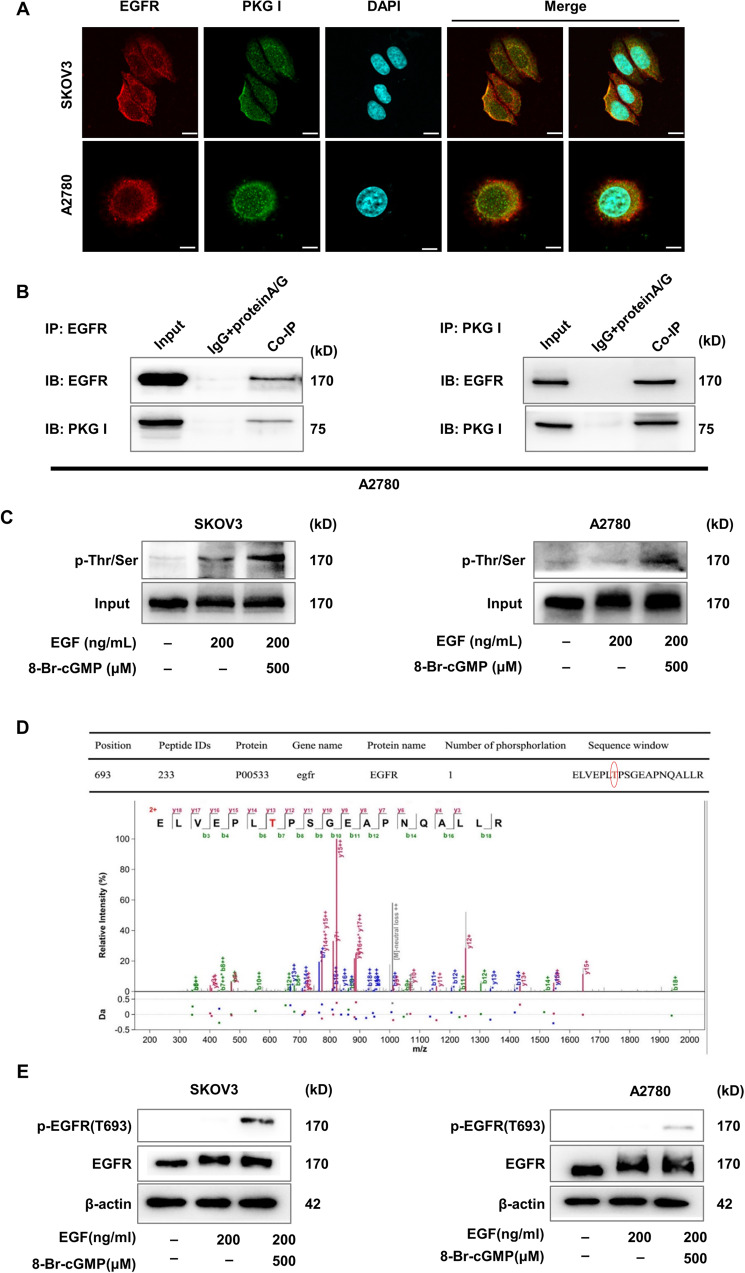
Activated PKG I bound with EGFR leading its serine and threonine phosphorylation. **A** Confocal laser scanning microscopy images on the expressions of EGFR and PKG I in SKOV3 and A2780 cells. Scale bar, 5 μm. **B** Co-IP assay revealed that PKG I bounded with EGFR in SKOV3 and A2780 cells. **C** Immunoprecipitation assay the serine and threonine phosphorylation of EGFR in SKOV3 and A2780 cells. **D** Mass spectrometry analysis identified that activated PKG I caused threonine 693 phosphorylation of EGFR. **E** Western blot analysis showed that activated PKG I caused phosphorylation of EGFR threonine 693 site in SKOV3 and A2780 cells.

Then we investigated the mechanisms underlying the tumor-suppressive effects of activated PKG I. The localization of EGFR and PKG I was visualized by confocal laser scanning microscopy. Subcellular localization analysis revealed that EGFR was located both in the cytomembrane and nucleus of EOC cells. PKG I was predominantly located in the cytomembrane and cytoplasm, but less in the nucleus of EOC cells. Overlapping fluorescence signals showed the co-localization of EGFR and PKG I in the cytomembrane but less in the nucleus of EOC cells (Fig. 5A). Furthermore, reciprocal Co-IP assay demonstrated that the PKG I and EGFR were presented in the EOC cells lysates (Fig. 5B). These results suggested that the PKG I bounded to EGFR in the EOC cells. PKG I is a serine and threonine kinase and activated by 8-Br-cGMP. Immunoprecipitation analysis showed that activated PKG I caused serine and threonine phosphorylation of EGFR in SKOV3 and A2780 cells (Fig. 5C). To identify the specific serine and threonine site, MS was performed to analyze the phosphorylation site on EGFR isolated from A2780 cells that treated with EGF (200 ng/mL) and 8-Br-cGMP (500 µM) for 24 h. It identified that T693 was the PKG I-specific phosphorylation site on EGFR (Fig. 5D). Western blot analysis further confirmed increased expressions of T693 phosphorylation of EGFR in SKOV3 and A2780 cells treated with EGF (200 ng/mL) and 8-Br-cGMP (500 µM) (Fig. 5E). These results confirmed that T693 of EGFR was the PKG I-specific phosphorylation site.

### Activation of PKG I inhibited EGF-induced EGFR/MAPK/ERK pathway

**Fig. 6:**
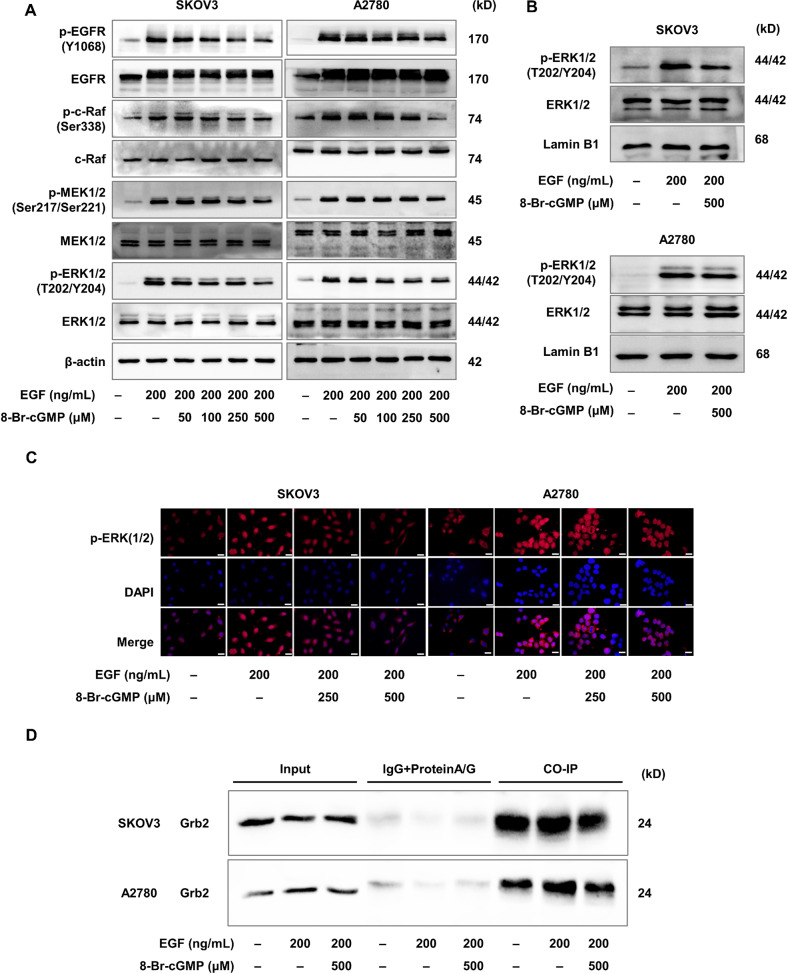
Activation of PKG I inhibited EGF-induced EGFR/MAPK/ERK pathway. **A** The activation of PKG I inhibited the phosphorylation levels of EGFR, c-Raf, MEK1/2, and ERK1/2 in SKOV3 and A2780 cells. Western blot (**B**) and immunofluorescence imaging (**C**) analysis the expression of nuclear phosphorylated ERK1/2 in SKOV3 and A2780 cells. Represent images were shown, respectively. Scale bar, 50 μm. **D** The Co-IP assay revealed that the 8-Br-cGMP-induced EGFR/MAPK/ERK pathway blockade via hampering the EGF-induced EGFR-Grb2-SOS1 binding.

**Fig. 7:**
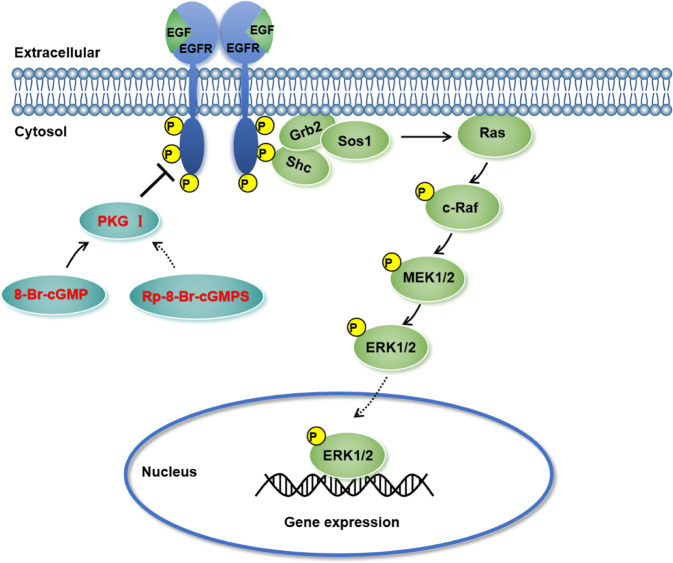
Schematic model of the activated PKG I attenuates EGF-induced EGFR/MAPK/ERK signaling in ovarian cancer. By binding to EGFR, the activated PKG I inhibit the expression of the EGFR/MAPK/ERK pathway and thereby decreases the expression of nuclear phosphorylated ERK1/2, ultimately leading to suppressed cell growth and metastasis in human EOC.

Finally, we investigated the role of activated PKG I in the EGFR-related signaling pathway. After the addition of EGF or 8-Br-cGMP, the total protein levels of EGFR, c-Raf, MEK1/2, and ERK1/2 remained unchanged. In SKOV3, the addition of EGF increased the phosphorylation of the EGFR/MAPK/ERK pathway-related proteins including the p-EGFR, p-c-Raf, p-MEK1/2, and p-ERK1/2 compared with the control group (Fig. 6A). In A2780 cells, the addition of EGF increased the levels of the p-EGFR, p-c-Raf, p-MEK1/2, and p-ERK1/2 compared with the control group (Fig. 6B). Their elevated these levels were attenuated by the 8-Br-cGMP supplement dose-dependently in SKOV3 and A2780 (Fig. 6A, B). The phosphorylated ERK1/2 would translocate to the nucleus and regulate the phosphorylation and activation of transcription factors inducing proliferation and migration. Indeed, the western blotting and immunofluorescence staining assay revealed that EGF treatment increased the expression of phosphorylated ERK1/2 in the nucleus of SKOV3 and A2780 cells (Fig. 6B, C). Whereas the 8-Br-cGMP supplement decreased the expression of nuclear phosphorylated ERK1/2 (Fig. 6B, C). The Co-IP assay revealed that the 8-Br-cGMP-induced EGFR/MAPK/ERK pathway blockade via hampering the EGF-induced EGFR-Grb2-SOS1 binding (Fig. 6D). Importantly, the suppression of phosphorylated EGFR and ERK1/2 induced by the activated PKG I was also abrogated by its inhibitor Rp-8-Br-cGMPS (Fig. 5). Collectively, these results suggested that the activated PKG I attenuated EGF-induced EGFR/MAPK/ERK signaling, leading to suppressed cell growth and metastasis in human EOC (Fig. 7).

## Discussion

In this study, we first observed higher expressions of EGFR and PKG I in EOC tissues and cells, but the level of phosphorylation of VASP was decreased. Then, PKG I activator 8-Br-cGMP, effectively inhibited EGF-induced EOC cell growth and metastasis in vivo and in vitro. Last, we demonstrated that activated PKG I interacted with EGFR and subsequently caused increased threonine 693 site phosphorylation and decreased tyrosine phosphorylation. This led to the decrease of phosphorylated downstream proteins of EGFR, including c-Raf, MEK1/2, and ERK1/2. Consequently, the nucleus translocation of phosphorylated ERK1/2 and related transcription activity was inhibited. This data suggested that elevated PKG I activity attenuated EGF-induced EOC cell proliferation and migration via the MAPK/ERK pathway, serving as a potential target for EOC treatment.

Ovarian cancer, especially EOC, is the most lethal gynecologic malignancy. It is essential to understand the underlying molecular mechanisms involved in the EOC progression to develop novel therapeutic approaches. It has demonstrated that the overexpressed EGFR has been considered as poor prognosis factor of EOC patients. Recently, growing evidence has shown that the cGMP⁄PKG pathway’s activation resulted in the inhibition of cell proliferation and induction of metastasis in cancer cells [21, 27–31]. EGFR is a well-characterized receptor tyrosine kinase and could be activated upon binding with its ligand like EGF. The upregulated tyrosine phosphorylation of EGFR induced an intracellular signal transduction cascade participating biological processes such as cell proliferation, differentiation, migration, adhesion, and angiogenesis [32]. Hence, we investigated whether the activation of the cGMP/PKG pathway also could inhibit EGF-induced EOC cell growth and metastasis.

In this study, we first found elevated expression of EGFR in EOC tissues and cell lines, which is in line with the previous findings [25]. Decreased PKG I levels have been confirmed in breast, liver, lung, and colon tumors [30] but its levels in ovarian cancer remained controversial. Wong et al. detected decreased PKG I expression in ovarian cancer cells [33], but we examined increased PKG I expression in EOC tissues and cell lines. However, the mechanism and whether the EGFR and PKG I was synergistically upregulated remains unclear, which would be an interesting point for further study. It is known that the cGMP activates the PKG I. The reduction in cGMP levels or its efficient bounding with PKG I will decline the activity of PKG I. By the ELISA assay, we examined downregulated cGMP levels in the ovarian cancer cells, which may account for the declined PKG I activity.

Then, the supplement of 8-Br-cGMP, a specific PKG I activator, suppressed the EGF-induced EOC cell proliferation, migration, and invasion in vitro. However, the PKG I agonist had no significant anti-ovarian cancer therapeutic effects in the absence of EGF, consisting with previous reports [21, 24]. Parashar reported small molecule inhibitors such as Gefitinib and Erlotinib blocked EGFR kinase activity to reduce ovarian cancer cells growth and peritoneal spread activity [34]. Activated PKG I also markedly attenuated the growth and metastasis in an EOC xenograft mouse model. Besides, the inhibition effect of activated PKG I in EGF-induced EOC cell growth and metastasis were reversed by the Rp-8-Br-cGMPS treatment, a PKG I inhibitor. Of interesting, the immunohistochemistry results showed that the expression of PKG I was high and there was no difference in nude mice or patient tissue with and without metastasis (Fig. 6). Thus, it looks like that the expression of PKG I has no effect on the tumor metastasis. More experiments were necessary to confirm the in *vivo* therapeutic effect of PKG I agonist. Overall, these findings suggested that activating PKG I may be a promising novel therapeutic strategy in EOC.

Last, we elucidated the underlying mechanisms to identify potential EOC therapeutic targets. We demonstrated that the PKG I interacted with EGFR and induced the increased serine and threonine phosphorylation of EGFR. MS analysis found that T693 of EGFR was the PKG I-specific phosphorylation site which is the same like PKG II. Activation of PKG I could inhibit the EGF-induced proliferation, invasion, and metastasis of EOC. We examined how PKG I impact on the tyrosine phosphorylation of EGFR and its downstream pathways. The MEK/ERK pathway is a major signaling pathway activated by EGFR signaling [23, 34]. The EGFR/MEK/ERK pathway has been reported to be involved in tumor progression in a variety of cancers [35, 36]. Here, our results showed that the activated PKG I decreased EGF-induced phosphorylation of EGFR (Y1068), c-Raf, MEK1/2, and ERK1/2 involved in the MEK/ERK pathway. Furthermore, translocation of p-ERK1/2 into the nucleus was decreased dramatically by the activated PKG I. Such effects were also reversed by the Rp-8-Br-cGMPS treatment. To our knowledge, we first identified the role of PKG I in inhibiting EGF-induced EOC progression. It has been reported that the activation of PKG I β is sufficient to inhibit cell growth and migration and induce apoptosis in human colon cancer cells. These effects are associated with the inhibition of the transcription of cyclin D1 and an increase in the expression of p21^CI^ [27]. Faranak et al. found that the activation of PKG by cGMP induced growth inhibition and apoptosis in MCF-7 and MDA-MB-468 breast cancer cell lines [22, 37]. Traci et al. reported that sGC stimulators and PDE5 inhibitors with increased cGMP reduced cell viability and apoptosis in head and neck cancer [21]. These studies suggested that activated PKG I attenuated tumors growth. On the other hand, anti-apoptotic actions of sGC/cGMP pathway have been also reported in lung and ovarian cancers, but we failed to observe the anti-apoptosis effect of PKG I in the SKOV3 and A2780 cells, suggesting that the outcome of PKG I activity depends on the cell-specific downstream effectors [23, 38, 39]. However, there was no report of activation PKG I on EGF-induced EGFR/ERK signaling pathway activation in EOC. Our study indicated that activated PKG I physically interacted with EGFR and induced its serine and threonine phosphorylation, leading its threonine 693 site phosphorylation, which thereby inhibited EGFR tyrosine phosphorylation and the EGFR/MEK/ERK signal pathway.

In conclusion, we demonstrated that the elevated activated PKG I by 8-Br-cGMP can interact with EGFR and disrupt its downstream MEK/ERK pathway related to cancer progression. The 8-Br-cGMP-PKG I-EGFR/MEK/ERK axis might serve as a novel a target for EOC treatment. Several drugs for treating non-malignant conditions act by increasing cGMP, which activates PKG approved by FDA [21]. These drugs might be worth trying to treat EOC in clinical trials.

## Supplementary information

Supplied Figure 1

Supplied Figure 2

Supplied Figure 3

Supplied Figure 4

Supplied Figure 5

Supplied Figure 6

Supplement figure legends

Supplied Table 1

## Data Availability

The materials described in the manuscript, including all relevant raw data, will be freely available to any researcher wishing to use them for non-commercial purposes, without breaching participant confidentiality.
