# Supplementary figures and images for: Increased endogenous PKG I activity attenuates EGF-induced proliferation and migration of epithelial ovarian cancer via the MAPK/ERK pathway
Source: Cell Death Dis. 2023 Jan 19;14(1):39. doi: 10.1038/s41419-023-05580-y (PMC9849337; doi:10.1038/s41419-023-05580-y)

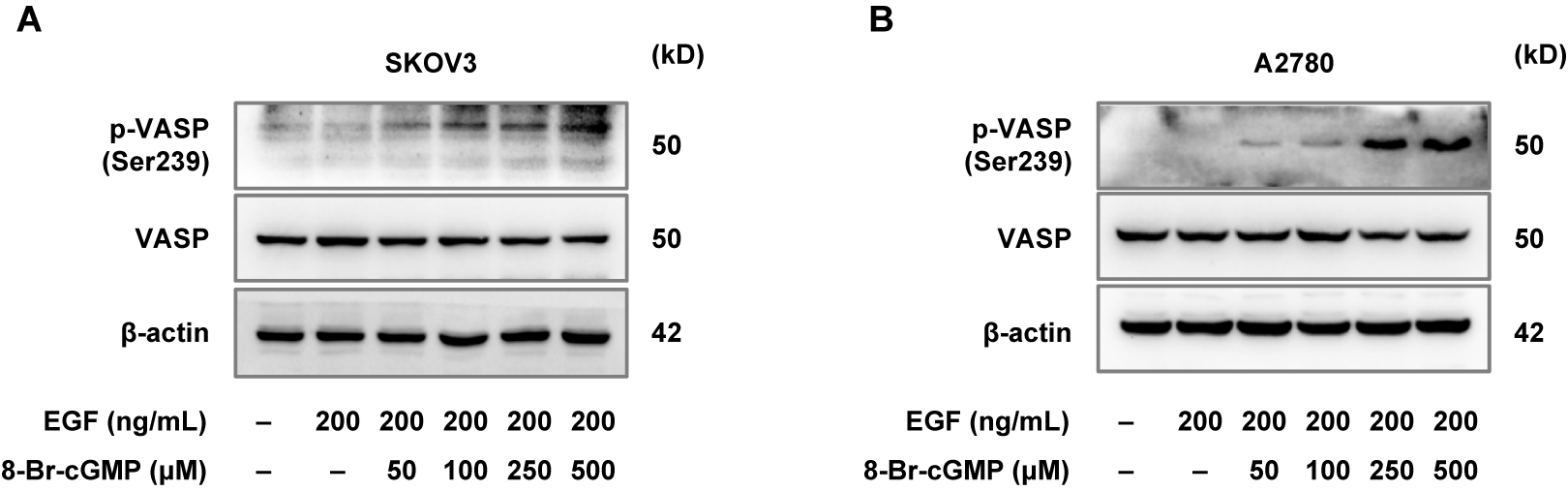

Supplement: Supplementary file 1 — Supplied Figure 1 [file 41419_2023_5580_MOESM1_ESM.tif]

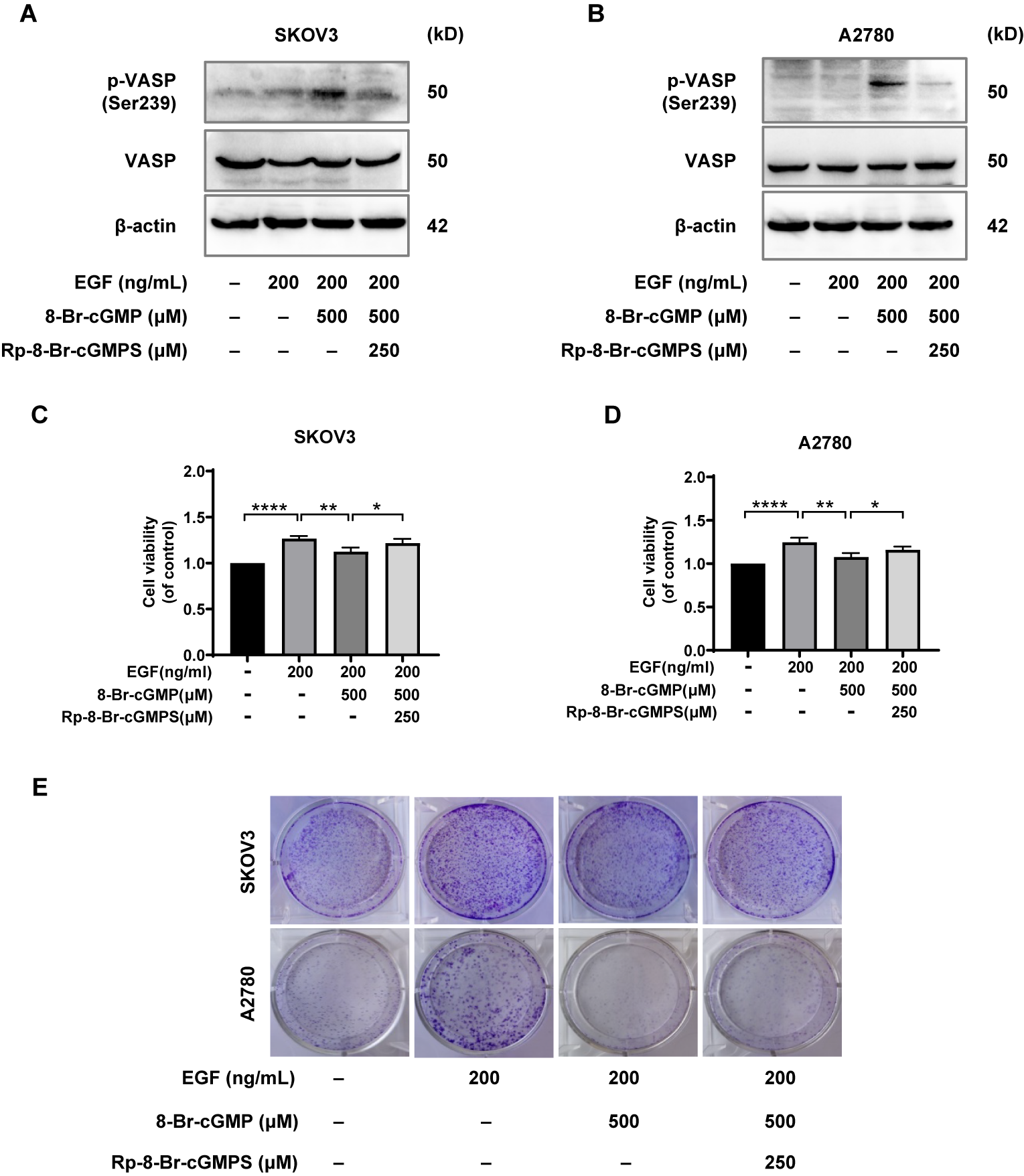

Supplement: Supplementary file 3 — Supplied Figure 3 [file 41419_2023_5580_MOESM3_ESM.tif]

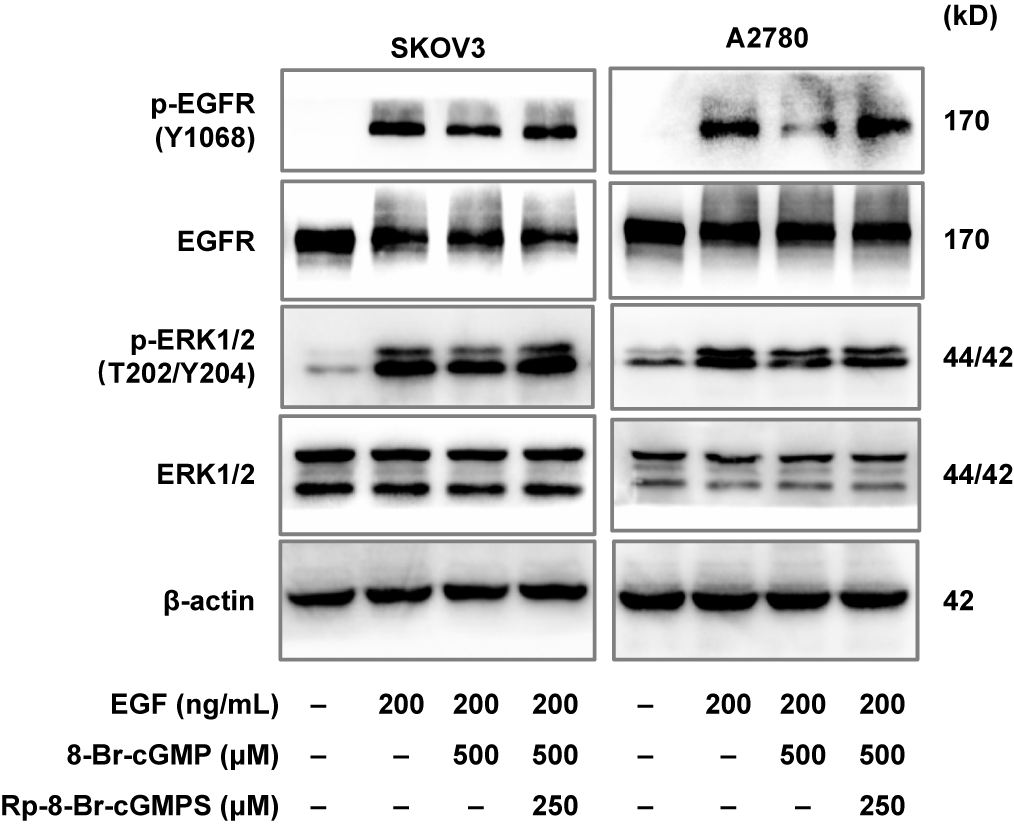

Supplement: Supplementary file 5 — Supplied Figure 5 [file 41419_2023_5580_MOESM5_ESM.tif]

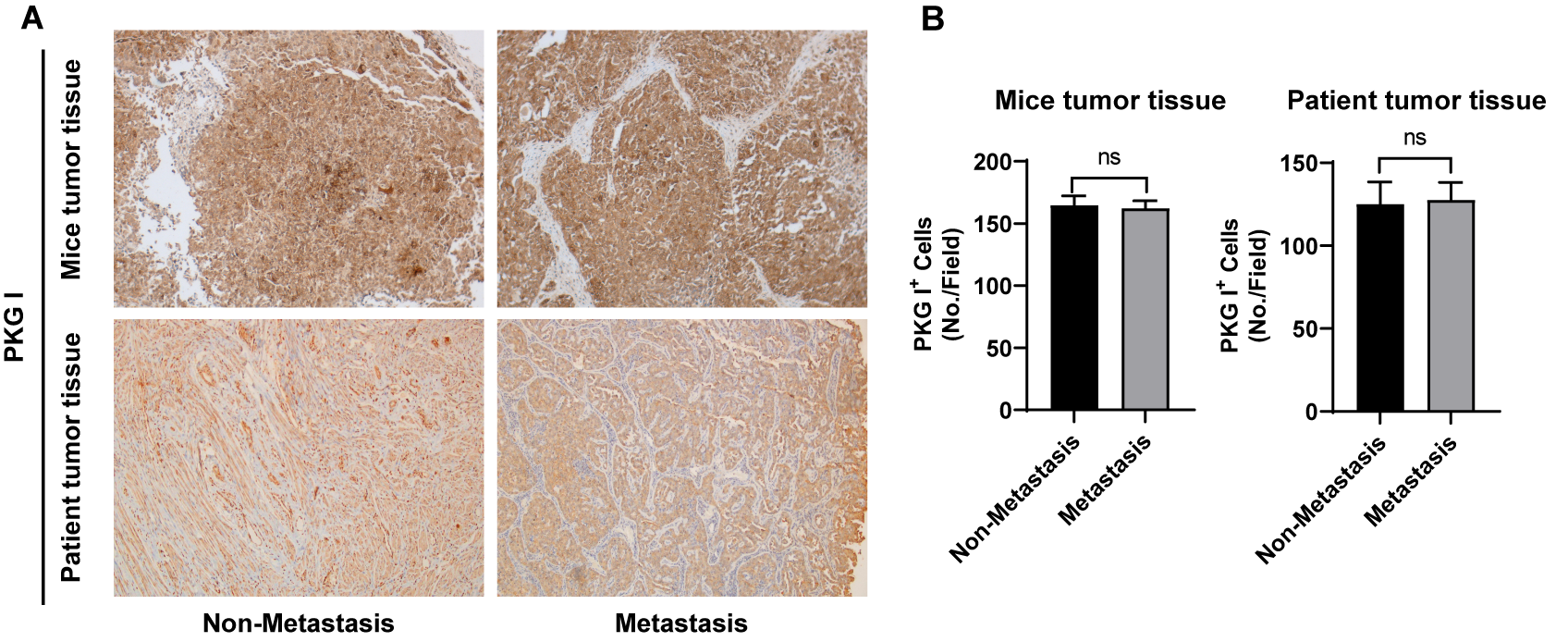

Supplement: Supplementary file 6 — Supplied Figure 6 [file 41419_2023_5580_MOESM6_ESM.tif]
