# Supplementary material for: Increased endogenous PKG I activity attenuates EGF-induced proliferation and migration of epithelial ovarian cancer via the MAPK/ERK pathway
Source: Cell Death Dis. 2023 Jan 19;14(1):39. doi: 10.1038/s41419-023-05580-y (PMC9849337; doi:10.1038/s41419-023-05580-y)
Supplement: Supplementary file 7 — Supplement figure legends [file 41419_2023_5580_MOESM7_ESM.docx]

**Supplied Figure 1. PKG I was activated by 8-Br-cGMP in ovarian cancer cells.** (**A, B**) The 8-Br-cGMP supplement, a specific PKG I activator, upregulated the expression of p-VASP in a dose-dependent manner.

**Supplied Figure 2. Activated PKG I also inhibited ovarian cancer proliferation, invasion, and metastasis in the absence of EGFR activation.** (**A**) CCK8 analysis the effect of 8-Br-cGMP on proliferation of EOC cells and normal ovarian cells in the absence of EGFR activation. Transwell analysis the effects of 8-Br-cGMP on migration (**B**) and invasion (**C**) of EOC cells in the absence of EGFR activation. (**D, E**) Effect of 8-Br-cGMP on apoptosis of EOC cells in the absence of EGFR activation. Significance was evaluated Ordinary two-way ANOVA analysis followed by Tukey’s multiple comparisons test in (A, D) ns *p* > 0.05; * *p* < 0.05 under indicated comparison.

**Supplied Figure 3. The Rp-8-Br-cGMPS treatment reversed the inhibition effect of activated PKG I and EOC cell proliferation.** (**A, B**) Western blot was used to elevated p-VASP expression with the Rp-8-Br-cGMPS treatment (250 µM) after 8-Br-cGMP (500 µM) supplement in SKOV3 and A2780 cells. (**C, D**) Quantitative data on the CCK-8 assay of SKOV3 and A2780 cells cultured with indicated EGF (200 ng/mL), 8-Br-cGMP (500 µM), and Rp-8-Br-cGMPS (250 µM). (**E**) Representative images on the colony assay of SKOV3 and A2780 cells cultured with indicated EGF (200 ng/mL), 8-Br-cGMP (500 µM), and Rp-8-Br-cGMPS (250 µM). The results were presented as mean ± SD (n=3). Significance was evaluated Ordinary two-way ANOVA analysis followed by Tukey’s multiple comparisons test in (E)* *p* < 0.05; ** *p* < 0.01; **** *p* < 0.0001 under indicated comparison.

**Supplied Figure 4. The Rp-8-Br-cGMPS treatment reversed the inhibition effect of activated PKG I on EOC cell migration and invasion.** (**A**) Wound healing assay images and quantitative data on SKOV3 and A2780 cells co-cultured with indicated EGF (200 ng/mL), 8-Br-cGMP (500 µM), and Rp-8-Br-cGMPS (250 µM). Scale bar, 100 μm. (**B, C**) Transwell assay was used to detect the inhibition effect of Rp-8-Br-cGMPS (250 µM) treatment on activated PKG I-induced migration and invasion in SKOV3 and A2780 cells. Scale bar, 50 μm. The results were presented as mean ± SD (n=3). Significance was evaluated Ordinary two-way ANOVA analysis followed by Tukey’s multiple comparisons test in (A, B, C)* *p* < 0.05; ** *p* < 0.01; *** *p* < 0.001; **** *p* < 0.0001 under indicated comparison.

**Supplied Figure 5. The Rp-8-Br-cGMPS treatment abrogated the suppression of phosphorylated EGFR and ERK1/2 in EOC cells induced by the activated PKG I.** Western blot assay on the expression of EGFR and ERK1/2 and their phosphorylation levels in the SKOV3 and A2780 cells cultured with indicated EGF (200 ng/mL), 8-Br-cGMP (500 µM), and Rp-8-Br-cGMPS (250 µM).

**Supplied Figure 6. The expression of PKG I in non-metastasis and metastasis mice and ovarian cancer patient’s tumor.** (**A, B**) Immunohistochemical assay on the expression of PKG I in non-metastasis and metastasis mice and ovarian cancer patient’s tumor. The results were presented as mean ± SD (n=5). Significance was evaluated by Student’s t test in (B) followed by Tukey’s multiple comparisons test in (D, E) ns *p* > 0.05 under indicated comparison.
