# Supplementary material for: Increased endogenous PKG I activity attenuates EGF-induced proliferation and migration of epithelial ovarian cancer via the MAPK/ERK pathway
Source: Cell Death Dis. 2023 Jan 19;14(1):39. doi: 10.1038/s41419-023-05580-y (PMC9849337; doi:10.1038/s41419-023-05580-y)
Supplement: Supplementary file 8 — Supplied Table 1 [file 41419_2023_5580_MOESM8_ESM.docx]

**Supplied Table 1**

| Antibodies | Vendor | Catalog number |
| --- | --- | --- |
| p-EGFR (T693) | Affinity Biosciences | AF3042 |
| Grb2 | Affinity Biosciences | DF4092 |
| p-ERK | Affinity Biosciences | AF1015 |
| EGFR | Affinity Biosciences | [AF6042](https://www.affbiotech.com/goods-1716-AF6042-EGFR_Antibody.html) |
| PKG I | Affinity Biosciences | [DF2677](https://www.affbiotech.com/goods-6758-DF2677-PRKG1_Antibody.html) |
| ERK1/2 | Affinity Biosciences | AF0155 |
| p-c-Raf(Ser338) | Affinity Biosciences | [AF3065](https://www.affbiotech.com/goods-1238-AF3065-Phospho_C_RAF_Ser338_Antibody.html) |
| c-Raf | Affinity Biosciences | [AF6065](https://www.affbiotech.com/goods-1725-AF6065-C_RAF_Antibody.html) |
| MEK1/2 | Affinity Biosciences | AF6385 |
| p-MEK1/2 | Affinity Biosciences | [AF3384](https://www.affbiotech.com/goods-1561-AF3384-Phospho_MEK1_2_Ser217_Ser218_Antibody.html) |
| p-VASP (Ser 239) | Affinity Biosciences | [AF3338](https://www.affbiotech.com/goods-1499-AF3338-Phospho_VASP_Ser239_Antibody.html) |
| VASP | Affinity Biosciences | [AF6337](https://www.affbiotech.com/goods-1941-AF6337-VASP_Antibody.html) |
| Ki-67 | Affinity Biosciences | [AF0198](https://www.affbiotech.cn/goods-897-AF0198-Ki67_Antibody.html) |
| MMP-9 | Affinity Biosciences | [AF5228](https://www.affbiotech.cn/goods-4535-AF5228-MMP9_Antibody.html) |
| Sos1 | Santa Cruz Biotechnology | sc-17793 |
| p-EGFR(Tyr1068) | Cell Signaling Technology | 3777 |
| β-actin | Proteintech Group | 81115-1-RR |
| p-Thr/ser | Abcam Biotechnology | ab17464 |
